# Biomarkers of systemic inflammation are associated with disease severity and metabolic syndrome in patients with hidradenitis suppurativa

**DOI:** 10.1016/j.jdin.2024.03.002

**Published:** 2024-03-15

**Authors:** Nikolaj Holgersen, Valdemar Wendelboe Nielsen, Nana Aviaaja Lippert Rosenø, Jacob P. Thyssen, Alexander Egeberg, Signe Holm Nielsen, Hans Christian Ring, Simon Francis Thomsen

**Affiliations:** a Department of Dermato-Venereology & Wound Healing Centre, Bispebjerg Hospital, Copenhagen, Denmark; b Department of Clinical Medicine, University of Copenhagen, Copenhagen, Denmark; c Nordic Bioscience, Herlev, Denmark; d Department of Biomedical Sciences, University of Copenhagen, Copenhagen, Denmark

**Keywords:** Biomarkers, disease severity, hidradenitis suppurativa, metabolic syndrome, systemic inflammation

## Abstract

**Background:**

Biomarkers associated with disease severity and comorbid metabolic syndrome (MetS) in patients with hidradenitis suppurativa (HS) are lacking.

**Objective:**

To identify biomarkers associated with disease severity and comorbid MetS in patients with HS.

**Methods:**

Data on hospital outpatients with HS were obtained through clinical examination and interviews. Indicators of systemic inflammation; C-reactive protein (CRP), erythrocyte sedimentation-rate (ESR), neutrophil/lymphocyte-ratio (NLR), platelet/lymphocyte-ratio (PLR), monocyte/lymphocyte-ratio (MLR), platelet/neutrophil-ratio (PNR), pan-immune-inflammation-value (PIV), and systemic-immune-inflammatory-index (SII), were calculated from blood samples.

**Results:**

Seven hundred patients were included; of those 444 (63.4%) and 256 (36.6%) were female and male, respectively, with a median age of 38.3 years (IQR = 27.9-51.0). Increasing CRP, ESR, NLR, PIV, and SII (*P* < .001) were significantly associated with increasing Hurley-stage and international hidradenitis suppurativa severity score system 4 (IHS4)-score in adjusted analysis. A doubling in CRP (OR 1.59 (1.36-1.85), *P* < .001), ESR (OR 1.39 (1.17-1.66), *P* < .001) and PIV (OR 1.41 (1.12-1.77) *P* = .002) was associated with MetS in adjusted analysis. ESR was the best estimator for severe IHS4-score (AUC = 0.72 (0.66-0.77), *P* < .001) and Hurley III (AUC = 0.79 (0.73-0.85), *P* < .001) whereas CRP was best for MetS (AUC = 0.67 (0.62-0.72), *P* < .001).

**Limitations:**

Patients in a hospital setting tend to have more severe disease.

**Conclusion:**

Biomarkers like CRP, ESR, and PIV measuring systemic inflammation were associated with disease severity and comorbid MetS in patients with HS.

**Capsule Summary**

• Biomarkers of systemic inflammation have previously been associated with cardiovascular disease, metabolic syndrome, and disease severity in hidradenitis suppurativa.
• Although these biomarkers can be used to estimate disease severity and metabolic syndrome in patients with hidradenitis suppurativa, the clinical implications yet remain unclear.

## Introduction

Hidradenitis suppurativa (HS) is a chronic, inflammatory skin disorder primarily involving apocrine gland-bearing areas of the skin, such as the axilla, groin, and anogenital area.^1^, ^2^, ^3^ It is characterized clinically by recurrent, painful nodules, abscesses, and tunnels (sinus tracts) with scarring. Reported HS prevalence rates vary from 0.1% to 4.0%,^3^^,^^4^ with a female predominance. Although the pathogenesis of HS is not fully understood, occlusion of hair follicles and a dysfunctional immune response leading to chronic inflammation has been suggested.^2^^,^^5^ Multiple risk factors in disease development and progression have been identified including smoking and obesity,^6^ although the exact consequences of these risk factors remain unclear. Several comorbidities also appear associated with HS, including metabolic syndrome (MetS)^7^, ^8^, ^9^ and cardiovascular disease and morbidity.^10^, ^11^, ^12^

Several biomarkers for systemic inflammation, including C-reactive protein (CRP), erythrocyte sedimentation rate (ESR), neutrophil/lymphocyte ratio (NLR), platelet/lymphocyte ratio (PLR), monocyte/lymphocyte ratio (MLR), platelet/neutrophil ratio (PNR), pan-immune-inflammation value (PIV), and systemic immune-inflammatory index (SII) are associated with cardiometabolic disease.^13^, ^14^, ^15^, ^16^, ^17^, ^18^, ^19^, ^20^, ^21^, ^22^ However use of these biomarkers in HS is poorly investigated, with few studies suggesting their potential use alongside clinical examination for evaluating HS severity.^23^, ^24^, ^25^ As HS severity evaluations suffer from interobserver variability,^26^ additional tools are needed to support clinicians. Since these biomarkers and indexes could be easily obtained and integrated into clinical evaluation of patients with HS, further investigations into their significance in assessing disease severity as well as their capacity to identify MetS are warranted.

In this study, we examined inflammatory biomarkers and their association with disease severity and MetS using a large hospital-based cohort of well-characterized patients with HS.

## Methods

### Demographic and clinical patient characteristics

Information on patient demographic factors (sex, age, body mass index [BMI]), smoking status, disease characteristics (Hurley stage and International HS severity score system [IHS4]),^27^ and presence of type 2 diabetes was obtained through clinical examination and interview of newly referred, consecutive outpatients with HS fulfilling the modified Dessau criteria^6^^,^^28^ at the Department of Dermato-Venereology and Wound Healing Centre, Bispebjerg Hospital, University of Copenhagen, Denmark.^29^

### Laboratory tests

The following blood test results were quantified: glucose, lipid levels (total cholesterol, low-density lipoprotein cholesterol, high-density lipoprotein cholesterol (HDL-c), triglycerides), haemoglobin, thrombocytes, and inflammatory markers; CRP, ESR, leucocytes, neutrophils, lymphocytes, basophils, eosinophils, and monocytes. For the present analyses, the latter was used to calculate indexes for systemic inflammation including NLR, PLR, MLR, PNR, PIV (calculated as (neutrophil count x platelet count x monocyte count)/lymphocyte count) and SII (calculated as [platelet count x neutrophil count]/lymphocyte count). Presence of MetS was for the purpose of this study defined as having at least 3 of the following criteria: obesity (BMI > 30 kg/m^2^), measured hypertension (systolic blood pressure > 130 mmHg and diastolic blood pressure > 85 mmHg), measured HDL-c < 1.0 mmol/L for males and < 1.3 mmol/L for females, measured elevated triglycerides > 1.7 mmol/L and/or presence of type 2 diabetes.^30^^,^^31^

### Statistical analysis

Statistical analyses were computed using IBM SPSS statistics version 28 (SPSS, Inc), GraphPad Prism version 10.0.0 for Windows, (GraphPad Software) and RStudio (Posit team (2023). RStudio: Integrated Development Environment for R. Posit Software, PBC). Variables were presented with either means and standard deviations or medians with IQR. Categorical variables were presented with numbers and percentages. Extreme outliers were excluded. For ANCOVA analysis, continuous variables were Log(e) transformed to achieve better approximation of normal distributions. Nonparametric Spearman’s correlations were calculated to assess significant correlations between variables. The 4 biomarkers with the best correlations to both disease severity and MetS were chosen for further analysis. The pROC-package^32^ (v.1.18.5) in RStudio was used to construct receiver operating characteristic (ROC) curves to assess the area under the curve (AUC) as well as optimal cut-off points for highest sensitivity and specificity for each biomarker in both disease severity and occurrence of MetS. Additionally, logistic regression fits were used to calculate prediction scores combining biomarkers in ROC-analysis, and the combination with highest AUC was reported. To further assess the relationship between biomarkers and occurrence of MetS, Box-Tidwell test was conducted to examine linearity of independent variables and log-odds before obtaining adjusted odds ratios (OR) using logistic regression with 95% CI. All results were considered of statistical significance with a *P* value <.05. Unless specified otherwise, percentages were listed as valid percentages.

## Results

### Clinical characteristics

We included a total of 700 adolescents and adults with HS. Of those, 444 (63.4%) were females and 256 (36.6%) males, with a median age of 38.3 years (IQR = 27.9-51.0) (Table I). Most were current (54.4%) or former (21.9%) smokers. In total 37.1% had Hurley stage I, 49.4% had Hurley stage II, and 13.4% had Hurley stage III. Median IHS4-score was 3.0 (IQR = 1.0-9.0), with 50.6% scoring as mild, 28.8% as moderate and 20.6% as severe. A total of 264 patients (39.4%) had hypertension, 262 (39.7%) had low HDL, 203 (30.8%) had elevated triglycerides (30.8%), 56 (8.0%) had type 2 diabetes, and 255 (36.9%) were obese. Median BMI was 27.9 kg/m^2^ (IQR = 23.7-32.6), with 30.7% being overweight (BMI 25-29.9 kg/m^2^) and 36.9% obese (BMI≥30 kg/m^2^). One hundred sixty patients (25.3%) had 3 or more of the above-mentioned criteria and were categorized as having MetS.

**Table I:** Demographic and clinical patient characteristics. Percentages calculated from available data

Patient characteristics	
Overall number of patients, *n* (%)	700 (100.0)
Age, years, median (IQR)	38.3 (27.9-51.0)
Sex, *n* (%)	
Female	444 (63.4)
Male	256 (36.6)
BMI [kg/m^2^], median (IQR)	27.9 (23.7-32.6)
Smoking status, *n* (%)	
Never	166 (23.7)
Previous	153 (21.9)
Current	381 (54.4)
Pack years, current smokers, median (IQR)	13.3 (5.7-28.0)
Hurley stage, *n* (%)	
I	260 (37.1)
II	346 (49.4)
III	94 (13.4)
IHS4-score, median (IQR)	3.0 (1.0-9.0)
IHS4 groups, *n* (%)	
Mild (≤3)	354 (50.6)
Moderate (4-10)	201 (28.8)
Severe (≥11)	144 (20.6)
MetS (≥3 criteria), *n* (%)	160 (25.3)
Measured hypertension	264 (39.4)
Low HDL	262 (39.7)
Elevated triglycerides	203 (30.8)
Diabetes, type 2	56 (8.0)
Obese (BMI ≥ 30 kg/m^2^)	255 (36.9)

*BMI*, Body mass index; *HDL*, High-density lipoprotein; *IQR*, interquartile range; *IHS4*, International Hidradenitis Suppurativa severity score; *MetS*, metabolic syndrome.

### Biomarkers associated with disease severity

**Fig 1:**
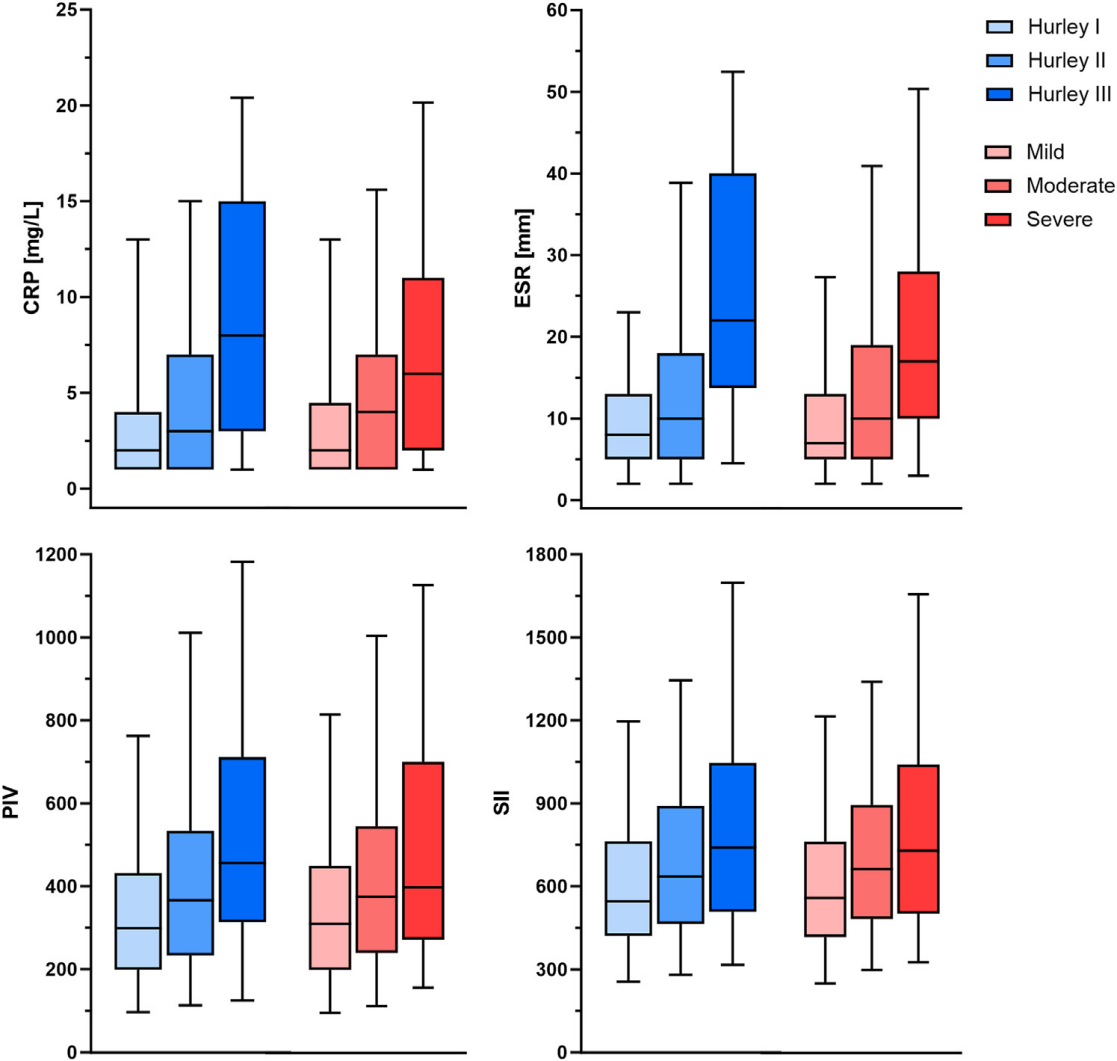
Biomarkers of systemic inflammation and their association with IHS4- and Hurley-stages. Data presented with 90% percentiles. *P* < .001 for ANCOVA between groups. *CRP*, C-reactive protein; *ESR*, erythrocyte sedimentation rate; *PIV*, pan-immune-inflammation-value; *SII*, systemic-immune-inflammatory-index.

In correlation analysis, CRP (rIHS4 = 0.420, rHurley = 0.352), ESR (rIHS4 = 0.379, rHurley = 0.422), PIV (rIHS4 = 0.253, rHurley = 0.311) and SII (rIHS4 = 0.211, rHurley = 0.261) were all moderately correlated to increasing disease severity (*P* < .001), whereas NLR (rIHS4 = 0.178, rHurley = 0.214), MLR (rIHS4 = 0.147, rHurley = 0.211) PNR (rIHS4 = −0.142, rHurley = −0.196), were all mildly correlated with increasing disease severity (*P* < .001). PLR was not significantly correlated (*P* > .05) (Fig 1, Supplementary Material, available via Mendeley at https://data.mendeley.com/datasets/rw56nvg9zy/1). In ANCOVA analysis adjusted for age, sex, current smoking, and BMI, increasing disease severity stage was significantly associated with increasing CRP, ESR, NLR, SIV, and SII. Significantly lower PNR was found with increasing disease severity for both IHS4-score (*P* < .001) and Hurley stage (*P* < .01). No significant differences in PLR or MLR were found within IHS4-groups or Hurley stage (*P* > .05) when adjusting for age, sex, smoking, and BMI.

**Fig 2:**
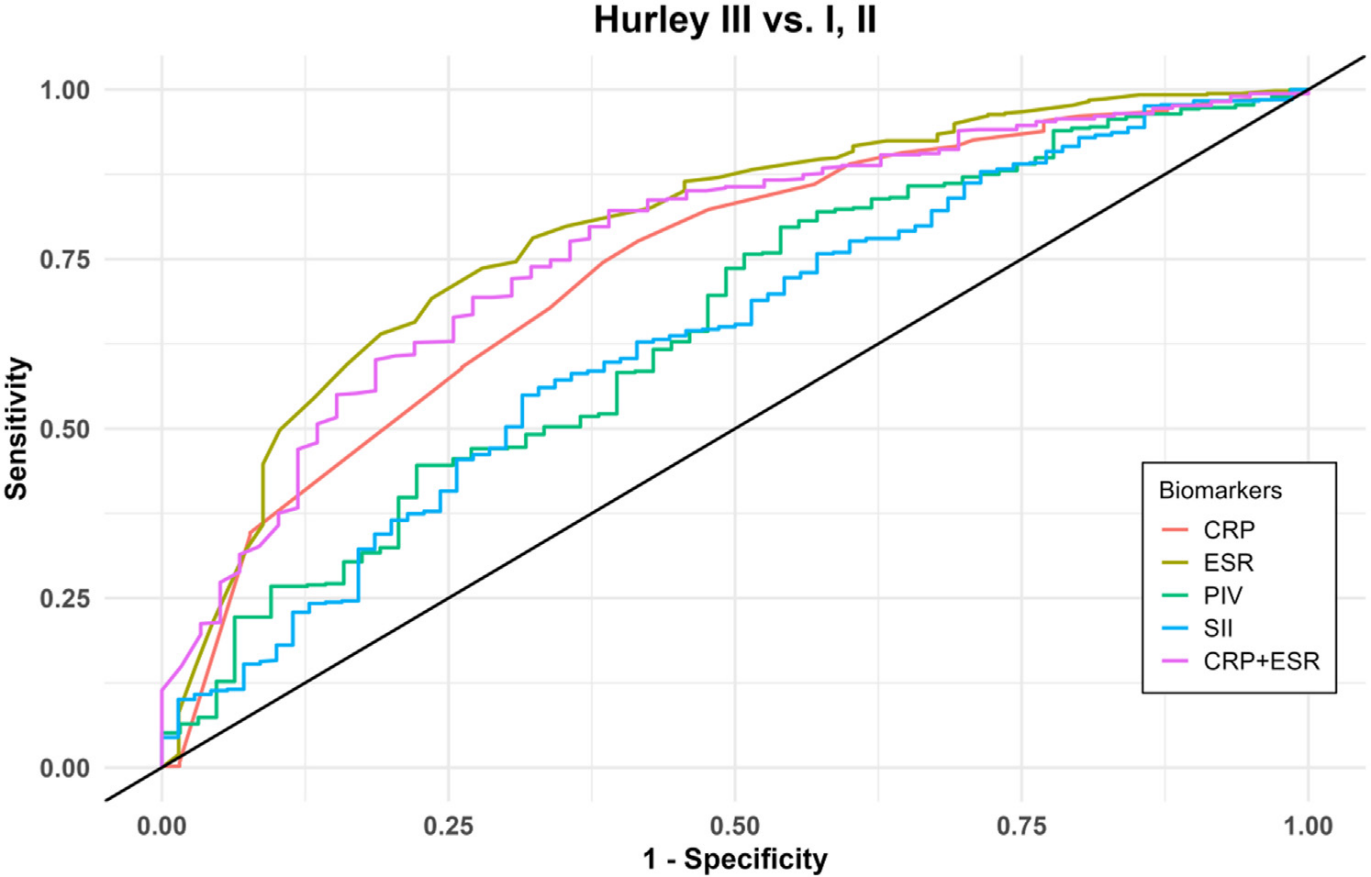
Receiver operating characteristics (ROC) curve for inflammatory biomarkers for Hurley III vs Hurley I, II. *CRP*, C-reactive protein; *ESR*, erythrocyte sedimentation rate; *PIV*, pan-immune-inflammation-value; *SII*, systemic-immune-inflammatory-index.

ROC-curves for the biomarkers with moderate correlations to disease severity (CRP, ESR, PIV, and SII) all showed significant results in discriminating between Hurley-as well as IHS4-groups (*P* < .001) (Table II). However, CRP and ESR were the best biomarkers in analysing disease severity, particularly when distinguishing between severe vs moderate and mild IHS4-score (CRP: AUC = 0.67 [0.61-0.72], *P* < .001, sensitivity = 0.76, specificity = 0.54. ESR: AUC = 0.72 [0.66-0.77], *P* < .001, sensitivity = 0.70, specificity = 0.65); and Hurley III vs Hurley I and II (CRP: AUC = 0.74 (0.67-0.80, *P* < .001, sensitivity = 0.78, specificity = 0.58. ESR: AUC = 0.79 [0.73-0.85], *P* < .001, sensitivity = 0.78, specificity = 0.67) (Fig 2, Supplementary Material, available via Mendeley at https://data.mendeley.com/datasets/rw56nvg9zy/1). When assessing the combination of biomarkers with highest explanatory value for severity assessment, a combination of CRP and ESR had the highest AUC for both mild vs moderate and severe IHS4-score (AUC = 0.66 [0.61-0.70], *P* < .001, sensitivity = 0.60, specificity = 0.68), severe vs mild and moderate IHS4-score (AUC = 0.70 (0.64-0.75), *P* < .001, sensitivity = 0.72, specificity = 0.60), Hurley I vs Hurley II and III (AUC = 0.63 [0.59-0.68], *P* < .001, sensitivity = 0.40, specificity = 0.83) and Hurley III vs Hurley I and II (AUC = 0.77 [0.70-0.83], *P* < .001, sensitivity = 0.82, specificity = 0.61).

**Table II:** AUC for individual biomarkers measured by receiver operating characteristics curve for IHS4-scores, Hurley-stage, and MetS

	IHS4 mild vs moderate, severe	IHS4 severe vs moderate, mild	Hurley I vs II, III	Hurley III vs I, II	MetS no vs yes
CRP					
AUC (95% CI)	0.65 (0.61-0.72)	0.67 (0.61-0.72)	0.62 (0.58-0.67)	0.74 (0.67-0.80)	0.67 (0.62-0.72)
*P* value	<.001	<.001	<.001	<.001	<.001
Cut-off	4.5	5.5	4.5	6.5	2.5
Sensitivity	0.48	0.76	0.44	0.78	0.76
Specificity	0.75	0.54	0.76	0.58	0.52
ESR					
AUC (95% CI)	0.66 (0.66-0.76)	0.72 (0.66-0.77)	0.63	0.79 (0.73-0.85)	0.63 (0.58-0.68)
*P* value	<.001	<.001	<.001	<.001	<.001
Cut-off	12.5	13.5	12.5	16.5	8.5
Sensitivity	0.54	0.70	0.48	0.78	0.71
Specificity	0.74	0.65	0.74	0.67	0.51
PIV					
AUC (95% CI)	0.61 (0.56-0.68)	0.62 (0.56-0.68)	0.62 (0.57-0.66)	0.65 (0.57-0.72)	0.60 (0.55-0.66)
*P* value	<.001	<.001	<.001	<.001	<.001
Cut-off	384.98	606.89	478.27	530.67	318.05
Sensitivity	0.50	0.85	0.38	0.80	0.74
Specificity	0.67	0.34	0.82	0.46	0.49
SII					
AUC (95% CI)	0.61 (0.56-0.67)	0.62 (0.56-0.68)	0.60 (0.55-0.64)	0.63 (0.56-0.70)	0.56 (0.51-0.61)
*P* value	<.001	<.001	<.001	<.005	<.05
Cut-off	702.67	636.98	557.18	636.98	609.0
Sensitivity	0.50	0.56	0.65	0.55	0.61
Specificity	0.68	0.63	0.52	0.68	0.51
CRP + ESR					
AUC (95% CI)	0.66 (0.61-0.70)	0.70 (0.64-0.75)	0.63 (0.59-0.68)	0.77 (0.70-0.83)	0.67 (0.62-0.72)∗
*P* value	<.001	<.001	<.001	<.001	<.001
Sensitivity	0.60	0.72	0.40	0.82	0.73
Specificity	0.68	0.60	0.83	0.61	0.59

*AUC*, Area under the curve; *CI*, confidence interval; *CRP*, C-reactive protein; *ESR*, erythrocyte sedimentation rate; *IHS4*, International Hidradenitis Suppurativa severity score system; *MetS*, Metabolic syndrome; *PIV*, pan-immune-inflammation-value; *SII*, systemic-immune-inflammatory-index.

∗ CRP + ESR + PIV.

### Biomarkers associated with MetS

**Fig 3:**
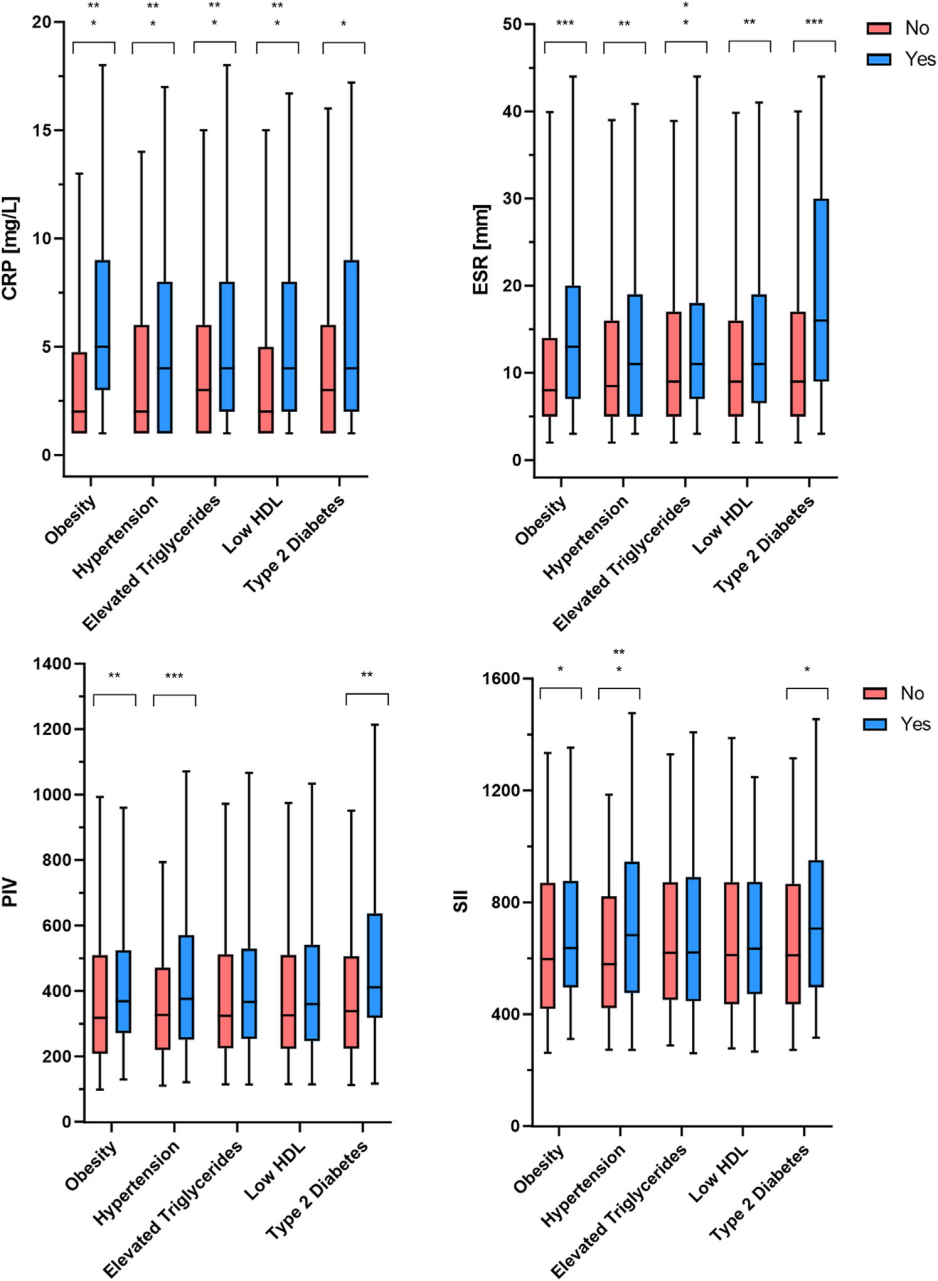
Levels of biomarkers of systemic inflammation and their association to MetS within different MetS-criteria. Data presented as medians with 90% percentiles. ∗*P* < .05, ∗∗*P* < .01, ∗∗∗*P* < .001. *CRP*, C-reactive protein; *ESR*, erythrocyte sedimentation rate; *MetS*, metabolic syndrome; *PIV*, pan-immune-inflammation-value; *SII*, systemic-immune-inflammatory-index.

CRP and ESR, respectively, were significantly elevated in obese patients with HS (*P* < .001 and *P* < .001), those with hypertension (*P* < .001 and *P* < .01), elevated triglycerides (*P* < .001 and *P* < .01), low HDL (*P* < .001 and *P* < .01), and type 2 diabetes (*P* < .05 and *P* < .001) (Fig 3). Selectively, PIV and SII were significantly elevated in patients with obesity (*P* < .01 and *P* < .05), hypertension (*P* < .001 and *P* < .001), and type 2 diabetes (*P* < .01 and *P* < .05). ROC-curves for individual biomarkers for presence of MetS were all significant (*P* < .05) (Table II). CRP (AUC = 0.67 [0.62-0.72], *P* < .001, sensitivity 0.75, specificity 0.52) and ESR (AUC = 0.63 [0.58-0.68], *P* < .001, sensitivity = 0.71, specificity = 0.51) were the best biomarkers for presence of MetS. Of all the biomarkers analyzed, a combination of CRP, ESR, and PIV (AUC = 0.67 [0.62-0.72], *P* < .001, sensitivity = 0.73, specificity = 0.59) were the best estimators for presence of MetS.

In logistic regression analysis, a doubling (log_2_ increase) in CRP (OR 1.59 [1.36-1.85], *P* < .001), ESR (OR 1.39 [1.17-1.66], *P* < .001), and PIV (OR 1.41 [1.12-1.77] *P* = .002) was significantly associated with presence of MetS compared to patients without, when adjusting for age, sex, current smoking, and disease severity (Supplementary Material, available via Mendeley at https://data.mendeley.com/datasets/rw56nvg9zy/1). SII was not significantly associated with presence of MetS (OR 1.22 [0.92-1.61], *P* = .164) when adjusting for these factors.

## Discussion

### Main findings

We found selected biomarkers of systemic inflammation (CRP, ESR, NLR, PIV, PNR, and SII, but not PLR and MLR) were associated with HS severity, even after adjusting for measured confounders. In ROC-analysis, CRP and ESR were the best estimators for disease severity assessment, and the combination of CRP and ESR was the most optimal combination. 25.3% of patients had MetS, and CRP, ESR, PIV, and SII were elevated within specific MetS criteria. CRP, ESR, and PIV were significantly associated with MetS in adjusted analysis albeit that their predictive values were only fair.

### Perspectives

NLR, SII, and PIV have previously been significantly correlated to increasing Hurley stage and Severity Assessment of HS score.^33^^,^^34^ Similarly, SII was elevated in patients with psoriasis and positively correlated with increasing Psoriasis Area Severity Index (PASI) score.^35^^,^^36^ To our knowledge, this is the first time that the association with disease severity is strong for both CRP, ESR, NLR, PNR, PIV, and SII in a large cohort of patients with HS, even when adjusting for possible confounders. However, these biomarkers only showed a moderate ability to differentiate between IHS4 and Hurley-stages, and the clinical implication for disease severity estimation from these biomarkers alone is probably limited. Considering that CRP and ESR emerged as the best estimators of severity across all groups, it raises the questions about the necessity for computing different inflammatory indices over using CRP and ESR which are routinely used biomarkers. While the predictive values of CRP, ESR, PIV, and SII were modest, their strong associations with Hurley stage and IHS4-scores suggest they could potentially function as surrogate measures for disease activity in assessing treatment efficacy and treatment response, but further investigation is necessary to confirm their potential utility.

We found a significant association of CRP, ESR, and PIV to MetS when adjusting for possible confounders. SII and NLR have previously been associated with MetS and insulin resistance and cardiovascular co-morbidity in patients with HS, although the latter was not statistically significant.^37^^,^^38^ Several scores, biomarkers, and imaging technologies exist for cardiovascular risk stratification,^39^ and novel approaches should be better or at least equally as effective as current methods. As the ability to estimate MetS from both CRP, ESR, PIV, and SII was ineffective with ROC-analysis, one could argue that these cannot stand alone in MetS risk stratification and prediction. Nevertheless, given the association between the biomarkers for systemic inflammation and MetS, they hold the potential to assist clinicians in evaluating the necessity of supplementary cardiovascular risk screening, as elevated levels of these biomarkers may serve as indicators of MetS in patients with HS.

### Strengths and limitations

As the demographic and clinical characteristics of our patients correspond well with existing literature,^4^ it enhances the generalizability of our findings. Patients were seen by the same group of physicians, which reduced interobserver variability, strengthening the findings of this study. A limitation of this study is that patients seen in a hospital setting tend to be more severely affected by their disease than patients seen in the primary care sector, which could reduce generalizability. Although this study finds an association between disease severity as well as MetS across several biomarkers, the included biomarkers are unspecific measurements of inflammation, and elevated levels could be a result of a concurrent disease unrelated to the patient’s chronic skin condition. Moreover, some definitions of MetS-criteria rely on waist circumference, but this information was unavailable in this study, and BMI was utilized as a substitute. Finally, to fully characterize the potential of inflammatory biomarkers for disease severity and cardiometabolic risk prediction, prospective studies are needed.

## Conclusion

Biomarkers of systemic inflammation were associated with disease severity and presence of MetS in patients with HS. The putative clinical implication of measuring these biomarkers remain unclear, and additional studies are needed to inform on their use.

## Conflicts of interest

None disclosed.
